# Inhibitory effects of polysaccharide extract from *Spirulina platensis* on corneal neovascularization

**Published:** 2009-09-24

**Authors:** Lingling Yang, Yao Wang, Qingjun Zhou, Peng Chen, Yiqiang Wang, Ye Wang, Ting Liu, Lixin Xie

**Affiliations:** State Key Laboratory Cultivation Base, Shandong Provincial Key Lab of Ophthalmology, Shandong Eye Institute, Qingdao, China

## Abstract

**Purpose:**

To assess the effects of polysaccharide extract from *Spirulina platensis* (PSP) on corneal neovascularization (CNV) in vivo and in vitro.

**Methods:**

PSP was extracted from dry powder of *Spirulina platensis*. Its anti-angiogenic activity was evaluated in the mouse corneal alkali burn model after topical administration of PSP four times daily for up to seven days. Corneal samples were processed for histochemical, immunohistochemical, and gene expression analyses. The effects of PSP on proliferation, migration, tube formation, and serine threonine kinase (AKT) and extracellular regulated kinase1/2 (ERK1/2) signaling levels in vascular endothelial cells were determined using 3-(4,5)-dimethylthiahiazo (-z-y1)-3, 5-di-phenytetrazoliumromide (MTT) and carboxyfluorescein succinimidyl ester (CFSE) labeling assays, wound healing assay, Matrigel tube formation assay, and western blot.

**Results:**

Topical application of PSP significantly inhibited CNV caused by alkali burn. Corneas treated with PSP showed reduced levels of platelet endothelial cell adhesion molecule (CD31) and stromal cell-derived factor 1 (SDF1) proteins, reduced levels of vascular endothelial growth factor (VEGF), matrix metalloproteinase-2 (MMP2), matrix metalloproteinase-9 (MMP9), SDF1, and tumor necrosis factor-alpha (TNF-α) mRNAs, and an increased level of pigment epithelium-derived factor (PEDF) mRNA. These are parameters that have all been related to CNV and/or inflammation. In human vascular endothelial cells, PSP significantly inhibited proliferation, migration, and tube formation in a dose-dependent manner. Furthermore, PSP also decreased the levels of activated AKT and ERK 1/2.

**Conclusions:**

These data suggest that polysaccharide extract from *Spirulina platensis* is a potent inhibitor of CNV and that it may be of benefit in the therapy of corneal diseases involving neovascularization and inflammation.

## Introduction

Under normal physiologic conditions, corneal tissue maintains its avascular transparent characteristics [1,2]. The molecular basis of the cornea’s avascularity has been shown to be the presence of soluble vascular endothelial growth factor (VEGF) receptor 1 [2], nonvascular VEGF receptor 3 expressed by corneal epithelium [3], the balance between angiogenesis promoting factors (such as VEGF) and inhibiting factors (such as pigment epithelium-derived factor [PEDF]) [4,5], and the systemic distributions and molecular interactions of VEGF and soluble vascular endothelial growth factor receptor 1 (sVEGFR1) [6], which was clarified well in the latest review of Qazi et al. [7]. Corneal neovascularization (CNV) is a major, sight-threatening complication of some ocular disorders. It can result from corneal infections, chemical injury, stromal ulceration, aniridia, and limbal stem cell deficiency. CNV not only interferes with normal corneal wound healing but also affects corneal graft survival after keratoplasty [1,4,8,9]. CNV results from an imbalance between angiogenic and anti-angiogenic factors [4]. It has been shown that CNV involves over-proliferation, migration, and capillary tube formation by endothelial cells [10]. Moreover, CNV, including that induced by alkali burn, is accompanied by abnormal matrix metalloproteinase activity and excess inflammation. Chemokines, which are crucial mediators of inflammatory and immune responses, also contribute to multiple aspects of CNV [11,12]. Therapeutic agents that antagonize multiple targets are therefore likely to be of benefit in the treatment of CNV-related diseases.

Spirulina is an unbranched, helicoidal, filamentous blue-green algae of the family *Oscillatoriaceae*. Spirulina has long been used in several countries as a supplement in human and animal food either as a health drink or in tablet form because of its alimentary value [13]. Toxicological studies [14] have shown that spirulina is safe for human consumption. In addition to its special alimentary benefits, researchers are currently turning attention to its potential usefulness in health care and clinical applications [15,16]. Several reports have shown that spirulina possesses anti-inflammatory [17], immunosuppressive [18,19], antioxidant [20], radioprotective [21], and renoprotective [22] properties.

In this study, we investigated the effects of polysaccharide extract from *Spirulina platensis* on CNV caused by alkali burn in mice as well as its anti-angiogenic effects on human vascular endothelial cells. The results show that PSP exhibits anti-angiogenic and anti-inflammatory properties in vivo and in vitro and suggest that PSP may be a novel natural agent for prevention and cure of inflammatory CNV-related diseases.

## Methods

### Preparation of polysaccharide extract from *Spirulina platensis*

Polysaccharides were extracted from *Spirulina platensis* as described previously. Briefly, dried *Spirulina platensis* was soaked in 95% (v/v) ethanol overnight and then torrefied. The mass was resuspended in NaOH (pH 10.0) solution and incubated at 80 °C for 4–6 h. After removal of debris by centrifugation, the liquid phase was collected and adjusted to pH 7.0 followed by precipitation with 5% trichloroacetic acid (TCA) at 4 °C overnight. The mixture was centrifuged, and the supernatant was precipitated with 5% TCA for another 3 h. After centrifugation, the supernatant was precipitated with ethanol (1 volume of supernatant/5 volume of ethanol) at 4 °C overnight. The precipitate, mainly containing *Spirulina* polysaccharides (PSP), was washed twice with acetone, lyophilized in a freeze-dryer, and stored at −20 °C. Before use, the PSP preparation was dissolved in normal saline and filtered through a 0.22 μm pore filtration membrane. The concentration of stock polysaccharides was measured using the anthrone-sulfuric acid method and was adjusted to 100 μg/ml using compound sodium chloride eye drops (Nanjing LiYe Pharmceutical Co. Ltd, Nanjing, China).

### Evaluation of alkali-induced corneal neovascularization

All animal experiments were performed in accordance with the guidelines of the Association for Research in Vision and Ophthalmology Statement for the Use of Animals in Ophthalmic and Vision Research. The alkali-induced CNV mouse model was generated by direct application of 3 μl of 1 N NaOH to both eyes of the mice for 30 s under general anesthesia with intraperitoneal ketamine and chlorpromazine. The burned eyes were then immediately rinsed with 20 ml of normal saline. PSP eye drops were applied topically (5 μl) to the burned eyes four times daily for seven consecutive days. Control animals were treated topically with compound sodium chloride eye drops without PSP. Eight mice were used for each group. On day 7, eyes were photographed under a slit-lamp, and CNV was quantified using a method for determining corneal angiogenesis [23]. All mice were sacrificed, and their eyes were collected for further examination.

### Immunohistochemistry

Eyeballs were either fixed with formalin or snap-frozen in optimal cutting temperature (OCT) compound (Sakura Finetechnical, Tokyo, Japan). Formalin-fixed, paraffin-embedded serial sections (4 μm) were deparaffinized by sequential washing with xylene followed by washing with descending series of ethanol and were then processed for hematoxylin and eosin (H&E) staining. For immunofluorescence staining, cryosections (6 μm) were prepared from OCT-embedded eyeballs and were fixed in ice-cold acetone for 10 min. The sections were blocked with 10% normal goat serum for 15 min and stained with phycoerythrin (PE)-conjugated anti-CD31 monoclonal antibody (mAb) (1:100; BD Biosciences Pharmingen, San Diego, CA) or mouse anti- stromal cell-derived factor 1 (SDF1) mAb (1:50; Santa Cruz Biotechnology, Inc., Santa Cruz, CA) overnight at 4 °C. Sections for SDF1 staining were washed and stained for 30 min at 37 °C with rhodamine-conjugated goat anti-mouse immunog|obulin G (IgG) secondary antibody (1:100; Santa Cruz). After counterstaining with 4,6-diamidino-2-phenylindole (DAPI), the stained sections were viewed under an Eclipse TE2000-U microscope (Nikon, Tokyo, Japan). Negative controls were performed by omitting primary antibodies.

### Real time reverse transcription polymerase chain reaction

Total RNA from mouse corneas was extracted using a NucleoSpin RNA II kit (Macherey-Nagel, Düren, Germany) and reverse transcribed using a PrimeScript RT Reagent kit (Takara, Shiga, Japan) following the manufacturers' instructions. Quantitative polymerase chain reaction (PCR) was performed using the SYBR green method with Real Master Mix SYBR Green (Tiangen Biotech, Beijing China). Reactions were performed in an ABI 7500 Detection System (Applied Biosystems, Foster City, CA) for 45 cycles at 95 °C for 15 s and at 60 °C for 60 s after initial incubation for 10 min at 95 °C. Gene-specific C_t_ values were standardized based on ribosomal protein L5 (RPL5) C_t_ values obtained for each cDNA. Each sample included triplicate sets, and the mean values of which were used to calculate the ratios of specific mRNA levels. The dissociation curve for each amplification reaction was generated to confirm the absence of nonspecific amplification. Gel electrophoresis was used to determine that amplified products were of the expected sizes. Specific primers used in this study are listed in Table 1.

**Table 1:** Primers used for real-time PCR

**Gene**	**Forward primer**	**Reverse primer**	**Product size (bp)**
*VEGF*	GAGCAGAAGTCCCATGAAGTG	CATGGTGATGTTGCTCTCTGA	213
*MMP2*	CCCGATCTACACCTACACCAA	AAACCGGTCCTTGAAGAAGAA	217
*MMP9*	CGTCGTGATCCCCACTTACTA	AAGATGAACGGGAACACACAG	237
*SDF1*	CAGTCAGCCTGAGCTACCGA	TCTTCAGCCGTGCAACAATC	126
*TNF-α*	AAGGGATGAGAAGTTCCCAAAC	CCTTGTCCCTTGAAGAGAACC	264
*PEDF*	GGTGCAGGCCCAGATGAA	ACGCCAAGGAGAAGGATGCT	81
*RPL5*	GGAAGCACATCATGGGTCAGA	TACACATCTTCATCTTCCTCCATT	70

The primer sequence and product size of the primers used in this study were provided in the table.

### Cell proliferation assay

Human umbilical vein endothelial cells (HUVECs, ATCC CRL-1730) were used for in vitro analyses. Cell proliferation was measured using both 3-(4, 5)-dimethylthiahiazo (-z-y1)-3, 5-di- phenytetrazoliumromide (MTT) and carboxyfluorescein succinimidyl ester (CFSE) labeling assays. For MTT assays, HUVECs were incubated in a medium with 0, 1, 5, 10, 50, or 100 μg/ml of PSP for 72 h followed by 4 h incubation with MTT. The MTT transformed crystals were dissolved in dimethyl sulfoxide, and absorbance at 490 nm was measured using a microplate reader (Molecular Devices, Sunnyvale, CA). Cell proliferation was also measured using the CFSE labeling assay as previously described [24]. CFSE is used to fluorescently label live cells and is equally partitioned to daughter cells during division and can be used to measure cell proliferation. Briefly, the cells were washed three times with PBS and incubated with 1 μM CFSE dye (CFDA SEM Cell Tracer kit; Molecular Probes, Eugene, OR) for 15 min. The cells were then washed again, incubated with a fresh medium containing 10% FBS, and seeded in six well plates at a density of 5×10^4^ cells/well. After 24 h, the medium was replenished with fresh medium containing 0, 10, 50, or 100 μg/ml of PSP. Cells were analyzed by flow cytometry (FACScalibur; BD Biosciences, Billerica, MA) 72 h later. Each setting was performed in triplicate.

### Cell migration

The effect of PSP on the migration of HUVECs was evaluated using a wound healing assay. Briefly, the cells were plated on 24 well culture plates. A scratch was made with a micropipette tip after confluence was reached. Cultures were then rinsed to remove detached cells and were incubated for 24 h with a medium containing various concentrations of PSP or with the solvent control. The scratches were photographed, and the cell migration rates were calculated. In each group, three duplicate wells were assayed, and each assay was conducted at least three times.

### In vitro tube formation assay

Thirty microliters of Matrigel™ Matrix (BD Biosciences, Bedford, MA) was dispensed in 96 well plates and allowed to polymerize for 1 h at 37 °C. HUVECs were seeded at a density of 1.5×10^4^ cells/well in media containing various concentrations of PSP (0, 10, 50, or 100 μg/ml). Cultures were incubated at 37 °C for 6 h, and digital images were captured for the observation of tube structures.

### Western blot analysis

The serine threonine kinase (AKT) and extracellular regulated kinase1/2 (ERK1/2) levels of HUVECs treated with or without 50 μg/ml of PSP were analyzed using western blots with antibodies against phosphorylated proteins. Briefly, cells harvested in sodium dodecyl sulfate-polyacrylamide gel electrophoresis (SDS–PAGE) buffer were sonicated. Protein components were separated on 10% SDS–PAGE gels for 1 h at 160 V followed by a transfer to nitrocellulose membrane. The blots were blocked in 5% non-fat dry milk for 1 h and incubated with primary antibody for 1 h at room temperature. After three washes with 10 ml of Tris-buffered saline Tween-20 (TBST), blots were incubated with a horseradish peroxidase-conjugated secondary antibody (Amersham Biosciences, Piscataway, NJ) and visualized via enzyme-linked chemiluminescence using the enhanced chemiluminescence (ECL) kit (Chemicon, Temecula, CA).

### Statistical analysis

Data are presented as mean±SD. The differences between control and experimental conditions were evaluated by SPSS 10.0 software (one-way ANOVA), and p<0.05 was considered significant.

## Results

### PSP inhibited corneal neovascularization in vivo

**Figure 1:**
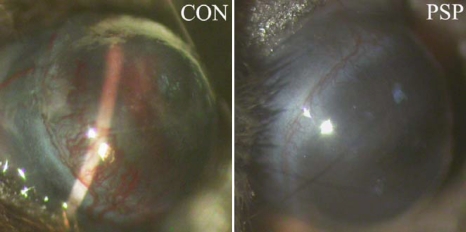
Macroscopic observation of corneal neovascularization in PSP-treated and control mice. PSP (100 μg/ml) or solvent control was used topically 4 times everyday on alkali-injured mice cornea for 7 days, images were taken with slit lamp. It can been seen clearly that in control group without PSP treated, the new vessel is predominant in conea, however which is very slight in cornea treated with PSP.

Topical application was used to evaluate the anti-angiogenic effects of PSP on alkali burn-induced CNV. In control mice, limbal vessels had sprouted into central corneas seven days after alkali burn. However, in the mice of the PSP-treated group, only a few vessels appeared near the corneal limbal area (Figure 1). Quantification assays showed that the average length of vessels in the PSP-treated group was 24% of that in the control group.

**Figure 2:**
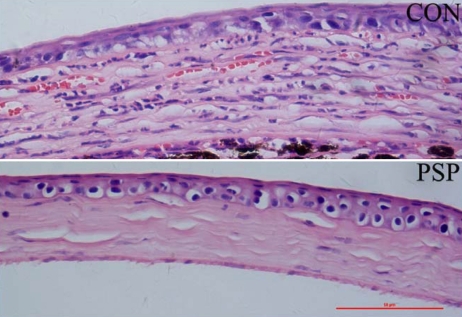
Histology of chemically burned corneas stained with H&E. H&E staining shows much more new vessels and mononuclear cells presented in control corneal stroma compared with PSP treated cornea. Three sections from different mice are shown in representative micrographs. Bar, 50 µm.

**Figure 3:**
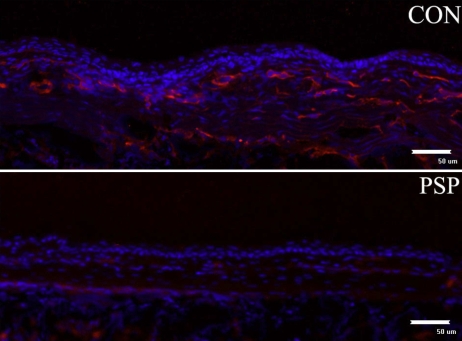
CD31 expression profile in alkali-burned mouse cornea. CD31-positive cells represent the vessel endothelium cells. The expression of stromal CD31 was decreased markedly in PSP treated cornea. Three specimens from different mice were studied in each group. Representative micrographs are shown in this figure. Bar, 50 µm.

**Figure 4:**
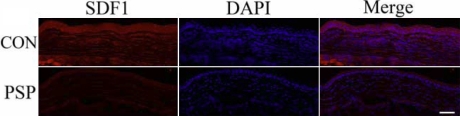
SDF1 expression profile in alkali-burned mouse cornea. The lymphocyte chemoattractant SDF-1 is an important inflammatory cytokine expressed in fibroblast in cornea. Topical PSP administration decreased the expression of SDF1 in cornea stroma effectively. Three specimens from different mice were studied in each group. Representative micrographs are shown. Bar, 50 µm.

**Figure 5:**
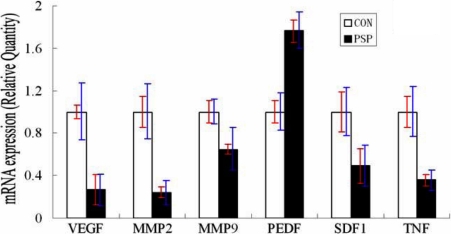
Changes in angiogenesis- and inflammation-related genes in PSP-treated CNV corneas. Compared with controls, the expressions of *VEGF*, *MMP2*, *MMP9*, *SDF1*, and *TNF* were reduced by 73%, 76%, 35%, 51%, and 64% respectively, while the expression of *PEDF* was increased by 76%. Red bars represent standard deviation, and blue bars represent a 95% confidence interval for the mean.

Histological H&E staining showed that the corneas of control mice possessed more new vessels and displayed more mononuclear cells and polymorphonuclear cell infiltration in the corneal stroma than did corneas treated with PSP (Figure 2). The levels of vascular endothelial cells and lymphocyte chemoattractant cytokine were examined by immunohistochemistry for CD31 and SDF1. The results showed that CD31 and SDF1 were prominent in the burned corneal stromas of control mice whereas PSP-treated corneas exhibited negligible staining (Figure 3 and Figure 4). Real-time PCR measurement of gene expression showed that both angiogenesis-related factors (VEGF, matrix metalloproteinase-2 [MMP2], and matrix metalloproteinase-9 [MMP9]) and inflammation-related factors (SDF1 and tumor necrosis factor-alpha [TNF-α]) were significantly repressed in PSP-treated corneas compared to control corneas whereas the anti-angiogenic factor, PEDF, was upregulated in PSP-treated corneas when compared to the control corneas (Figure 5). The expression level of RPL5 was similar in each group. Overall, mice treated with PSP showed markedly less severe corneal neovascularization as well as less severe inflammation and dropsy when compared with control mice that received no therapy.

### PSP inhibited the proliferation, migration, and tube formation of endothelial cells

**Figure 6:**
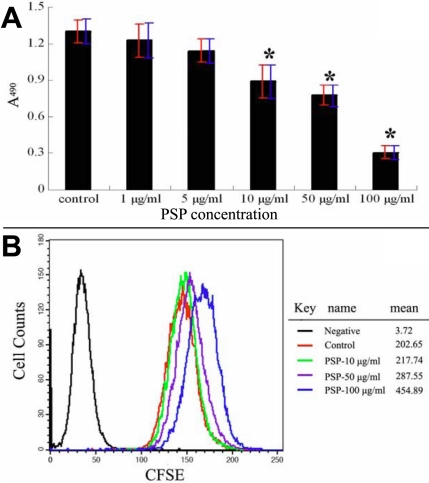
The effects of PSP on HUVEC proliferation. **A**: MTT assay. PSP could inhibit HUVECs proliferation significantly and dose-dependently. Red bars represent standard deviation, and blue bars represent a 95% confidence interval for the mean. **B**: CFSE labeling assay. The result confirmed the inhibition effect of PSP on HUVEC proliferation.

**Figure 7:**
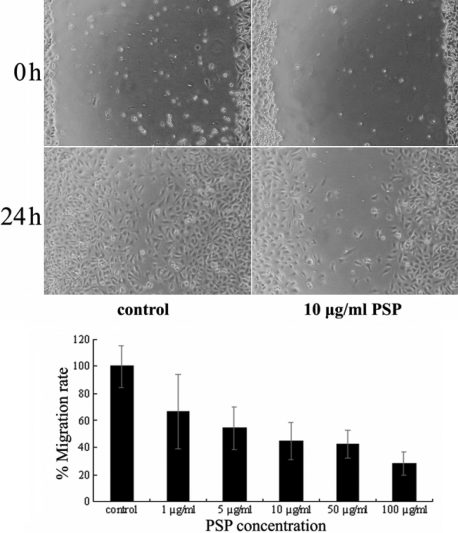
The effects of PSP on the migration of HUVECs. Representative photographs of migrating cells in corneas that received either control or PSP treatment are shown in the upper panel. In the lower panel, the migration rate in the control group was arbitrarily set at 100%.

**Figure 8:**
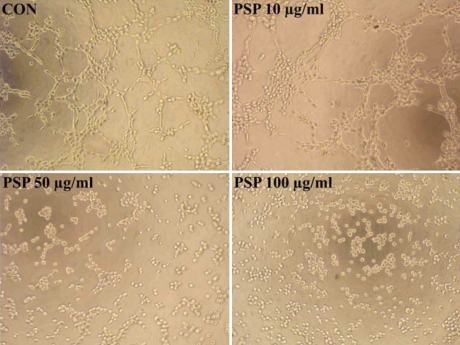
Effect of PSP on HUVEC tube formation. HUVECs were plated on the surface of Matrigel in complete media with 10, 50, or 100 µg/ml of PSP or without PSP, and tube formation was evaluated 6 h later. The angiogenic stuctures was inhibited by PSP obviously especially above concentration of 50 µg/ml. One representative experiment of three separate assays is shown.

Proliferation, migration, and tube formation by vascular endothelial cells play critical roles in corneal neovascularization. We therefore evaluated the effects of PSP on the cellular properties of HUVECs in vitro. MTT assays revealed that PSP inhibited the proliferation of HUVECs in a dose-dependent manner. The inhibitory effect was significant when concentrations above 10 μg/ml (p<0.05) were applied for 72 h (Figure 6A). CFSE staining analysis confirmed that the inhibitory effects of PSP on the proliferation of endothelial cells were mediated by reductions in the rate of cell division (Figure 6B). The wound healing assay showed that PSP had a significant, dose-dependent inhibitory effect on cellular migration (Figure 7). Furthermore, vascular endothelial cells seeded on the surface of solidified Matrigel could form capillary-like tube structures. When used above 10 μg/ml, PSP significantly reduced tube formation. No tube-like structures were seen when the PSP concentration was above 50 μg/ml (Figure 8).

### PSP inhibited AKT and ERK1/2 phosphorylation in endothelial cells

**Figure 9:**
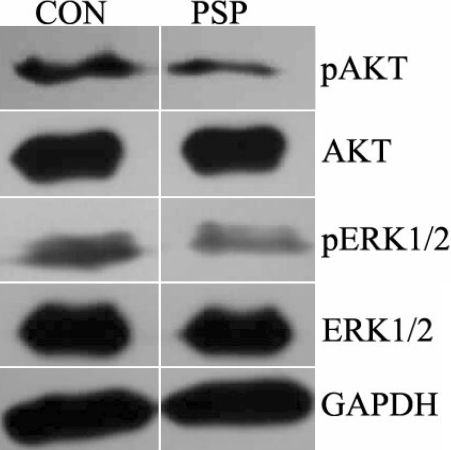
The effects of PSP on AKT and ERK1/2 signaling. The protein expression of phosphorylation and total AKT and ERK1/2 were assessed using total cellular protein lysates. PSP significantly reduced the level of phosphorylation of both AKT and ERK1/2 in HUVECs, although it had no effect on the total amount of AKT or ERK1/2 protein.

To determine whether treatment with PSP affects the signaling of endothelial cells through AKT or ERK1/2 pathways, HUVECs were treated with PSP (50 μg/ml) for 72 h and western blot assays were performed. As shown in Figure 9, PSP significantly reduced the level of phosphorylation of both AKT and ERK1/2 in HUVECs, although it had no effect on the total amount of AKT or ERK1/2 protein.

## Discussion

Ocular neovascularization is closely associated with local inflammation, and many chemokines and cytokines are involved in this process. Current therapies for CNV such as surgery, laser photocoagulation, and medication have limitations and complications. Application of natural angiogenic inhibitors could be a promising alternative or possible complementary therapy in management of CNV. In fact, several natural products with anti-angiogenic properties have shown to affect CNV in experimental animal models. These agents include genistein [25], shark cartilage [26], curcumin [27,28], and propolis extract [29]. In the present study, we demonstrated anti-angiogenic and anti-inflammation properties of polysaccharides from *Spirulina platensis* using the alkali-burn CNV model. We further confirmed that the anti-angiogenic effects of PSP were mediated by interference with the proliferation, migration, and tube formation of vascular endothelial cells in vitro (Figure 6, Figure 7, and Figure 8). Moreover, PSP dramatically decreased the levels of phosphorylated AKT and ERK1/2 in endothelial cells. Both of these protein kinases are involved in the angiogenic process [30-35].

Several animal models have been established for the purpose of studying the role of pathologic angiogenesis in corneal diseases including corneal micropocket, corneal suture, burn and chemical cornea models, intrastromal injection of proangiogenic factor, and partial limbal deficiency models. In these models, chemical corneal treatment is often used because it closely mimics the complex nature of the human disease and because the inflammatory response is the important component for neovascularization in most of these models. The corneal micropocket model is usually used to study the influence of specific molecules and proteins in angiogenesis [36,37]. Considering the known anti-inflammatory effects of spirulina [17] and the simulation of human ocular disease, we selected the alkali burn induced corneal neovascularization model to perform our study.

Topical application of angiogenic inhibitors is an advantageous route for prevention and treatment of CNV because it is non-invasive and has minimal systemic adverse effects [38-41]. The present study confirmed the effectiveness of PSP via topical application in CNV. Topical application of 100 μg/ml PSP four times daily significantly prevented alkali burn-induced CNV as confirmed by morphological observation and histochemical and immunohistochemical staining (Figure 1, Figure 2, and Figure 3).

To detect the effects of PSP on factors responsible for angiogenic or anti-angiogenic activity, we first selected VEGF, MMP2, and MMP9, which are major factors contributing to CNV, and PEDF, a typical anti-angiogenic factor, for our study [1,5,9]. As shown in Figure 5, PSP suppressed the expression of VEGF, MMP2, and MMP9 and stimulated the expression of PEDF, suggesting that PSP may inhibit CNV by downregulating the expression of angiogenic factors and upregulating the expression of the anti-angiogenic factor.

For the detection of possible anti-inflammatory effects of PSP on alkali-burned corneas, the inflammation factors SDF1 and TNF-α were selected for study. The SDF1/CXCR4 ligand/receptor pair is an important contributor to several types of ocular neovascularization [42]. TNF-α is known to be one of the key regulators of inflammation and can mediate angiogenesis [43]. In corneas, the infiltration of SDF1-positive inflammatory cells (Figure 4) and the expression of SDF1 and TNF-α RNA (Figure 5) were significantly depressed by PSP. In addition, we demonstrated through in vitro studies that PSP had significant inhibitory effects on HUVEC proliferation, migration, and tube formation ability (Figure 6, Figure 7, and Figure 8), which are the three key events that contribute to CNV. We propose that PSP inhibited CNV by directly interfering with endothelial cell behavior and by indirectly inhibiting production of inflammatory factors that regulate angiogenic properties.

In this assay, we found PSP treatment could dramatically decrease the amount of phosphorylated AKT and ERK1/2 in HUVECs, implying that the potential molecular mechanism of PSP inhibition of CNV may be partly attributed to its inhibition effect on the activation of AKT and ERK1/2 phosphorylation. However, PSP may also regulate other important factors involved in angiogenesis such as decreasing MMPs and VEGF by suppressing extracellular MMP inducer (EMMPRIN) expression [44-47], which should be clarified in future research.

PSP has been proven to possess multiple bioactivities including inhibition of tumor invasion and antiviral, antioxidant, chemoprotective and radioprotective properties [21,48-51]. To our knowledge, this study demonstrates for the first time that PSP has a strong inhibitory effect on inflammation-induced corneal neovascularization, which suggest the potential use of PSP in the treatment of inflammatory neovascularization-related corneal diseases. At present, the commonly used drugs to inhibit neovascularization in clinical are Avastin and Lucentis, which has been proven successful to treat choroidal neovascularization in age-related and myopic macular degeneration [52,53]. But considering the price, Avastin and Lucentis are too expensive for lower-income patients. PSP may be become a far cheaper alternative drug for the therapy of corneal neovascularization, although many basic and clinical trials must be first conducted to confirm its clinical efficacy and safety before its widespread clinical application. Further studies should focus on the possible toxicity and side effects of PSP associated with potential ocular applications.
